# Supplementary material for: Accelerometer-Measured Physical Activity Levels and Patterns Vary in an Age- and Sex-Dependent Fashion among Finnish Children and Adolescents
Source: Int J Environ Res Public Health. 2022 Jun 6;19(11):6950. doi: 10.3390/ijerph19116950 (PMC9180141; doi:10.3390/ijerph19116950)
Supplement: Supplementary file 1 [file ijerph-19-06950-s001.zip › FigS1. Relationship between oxygen consumption and mean amplitude deviation for adults and children.pdf]

Figure S1. Relationship between oxygen consumption and mean amplitude deviation for adults and children

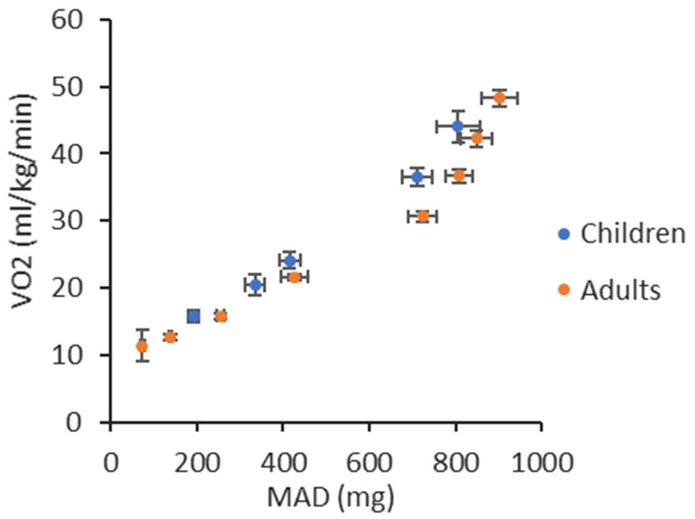


*The error bars represent the 95 % confidence intervals.
