# Supplementary material for: Accelerometer-Measured Physical Activity Levels and Patterns Vary in an Age- and Sex-Dependent Fashion among Finnish Children and Adolescents
Source: Int J Environ Res Public Health. 2022 Jun 6;19(11):6950. doi: 10.3390/ijerph19116950 (PMC9180141; doi:10.3390/ijerph19116950)
Supplement: Supplementary file 1 [file ijerph-19-06950-s001.zip › TabS1. Participants hourly step counts by sex and tertiles and p-values for tertile differences.pdf]

Table S1. Participants' hourly step counts by sex and tertiles and p-values for tertile differences.

		BOYS				GIRLS			
		step tertiles			**p-value (Kruskall-Wallis test)**	step tertiles			**p-value (Kruskall-Wallis test)**
	time of the day	least active	middle	most active		least active	middle	most active	
step count (hourly avarage)	7	541	571	673	**0.034**	454	591	634	**0.004**
	8	619	722	928	**<0.001**	559	712	786	**<0.001**
	9	624	774	972	**<0.001**	512	618	713	**<0.001**
	10	607	800	974	**<0.001**	513	612	728	**<0.001**
	11	671	903	1111	**<0.001**	561	663	836	**<0.001**
	12	671	888	1105	**<0.001**	554	656	838	**<0.001**
	13	672	863	1079	**<0.001**	563	722	905	**<0.001**
	14	633	852	1134	**<0.001**	576	759	975	**<0.001**
	15	476	756	1077	**<0.001**	481	649	889	**<0.001**
	16	498	783	1183	**<0.001**	441	660	933	**<0.001**
	17	565	918	1426	**<0.001**	479	721	1131	**<0.001**
	18	582	1045	1537	**<0.001**	511	764	1237	**<0.001**
	19	501	921	1356	**<0.001**	482	742	1065	**<0.001**
	20	339	560	922	**<0.001**	342	479	721	**<0.001**
	21	216	338	533	**<0.001**	228	349	477	**<0.001**
	22	165	227	325	**<0.001**	198	249	328	**<0.001**
	23	131	216	284	**0.015**	114	247	246	**<0.001**
step count on weekdays (hourly avarage)	7	574	614	686	**0.090**	477	609	666	**0.002**
	8	652	768	930	**<0.001**	575	719	808	**<0.001**
	9	664	805	967	**<0.001**	531	641	701	**<0.001**
	10	642	832	927	**<0.001**	530	610	686	**<0.001**
	11	713	931	1085	**<0.001**	574	681	777	**<0.001**
	12	720	913	1094	**<0.001**	583	673	786	**<0.001**
	13	723	911	1050	**<0.001**	593	756	884	**<0.001**
	14	669	863	1094	**<0.001**	616	800	974	**<0.001**
	15	485	724	1045	**<0.001**	495	654	873	**<0.001**
	16	508	782	1190	**<0.001**	446	672	950	**<0.001**
	17	596	980	1502	**<0.001**	495	762	1195	**<0.001**
	18	598	1136	1647	**<0.001**	544	813	1341	**<0.001**
	19	525	992	1441	**<0.001**	512	776	1136	**<0.001**
	20	351	603	997	**<0.001**	335	498	768	**<0.001**
	21	221	359	586	**<0.001**	235	339	509	**<0.001**
	22	183	234	319	**<0.001**	220	269	372	**<0.001**
	23	103	234	281	**0.010**	143	213	280	**0.007**
step count on weekends (hourly avarage)	7	119	164	545	**0.005**	80	258	300	**0.008**
	8	264	398	805	**<0.001**	287	444	508	**<0.001**
	9	356	590	1090	**<0.001**	377	461	822	**<0.001**
	10	454	684	1277	**<0.001**	426	663	959	**<0.001**
	11	527	784	1247	**<0.001**	506	619	1034	**<0.001**
	12	499	812	1145	**<0.001**	439	614	1034	**<0.001**
	13	501	730	1170	**<0.001**	451	610	966	**<0.001**

14	524	824	1233	**<0.001**	448	616	958	**<0.001**
15	458	860	1192	**<0.001**	438	638	972	**<0.001**
16	479	802	1203	**<0.001**	431	622	902	**<0.001**
17	457	731	1147	**<0.001**	422	609	876	**<0.001**
18	507	753	1167	**<0.001**	424	594	914	**<0.001**
19	432	691	1033	**<0.001**	385	608	832	**<0.001**
20	316	463	728	**<0.001**	366	422	602	**<0.001**
21	219	281	351	**0.004**	199	370	450	**<0.001**
22	137	231	322	**<0.001**	143	236	316	**<0.001**
23	158	227	212	**0.041**	72	288	253	**<0.001**
