# Supplementary material for: Accelerometer-Measured Physical Activity Levels and Patterns Vary in an Age- and Sex-Dependent Fashion among Finnish Children and Adolescents
Source: Int J Environ Res Public Health. 2022 Jun 6;19(11):6950. doi: 10.3390/ijerph19116950 (PMC9180141; doi:10.3390/ijerph19116950)
Supplement: Supplementary file 1 [file ijerph-19-06950-s001.zip › TabS2. Participants hourly LPA minutes by sex and tertiles and p-values for tertile differences.pdf]

Table S2. Participants' hourly LPA minutes by sex and tertiles and p-values for tertile differences

		BOYS				GIRLS			
		step tertiles			**p-value (Kruskall-Wallis test)**	step tertiles			**p-value (Kruskall-Wallis test)**
	time of the day	least active	middle	most active		least active	middle	most active	
LPA minutes (hourly average)	7	5,0	6,9	8,7	**0,272**	5,9	8,8	8,9	**0,128**
	8	12,1	13,7	13,0	**0,665**	13,6	12,9	13,9	**0,468**
	9	11,4	11,1	12,0	**0,112**	10,7	11,3	10,9	**0,071**
	10	9,7	9,6	9,6	**0,822**	8,0	8,9	9,0	**0,000**
	11	9,0	9,5	9,5	**0,057**	7,8	8,4	8,8	**0,000**
	12	9,2	9,5	9,9	**0,027**	8,1	8,9	9,2	**0,000**
	13	9,3	9,9	9,8	**0,008**	8,4	8,9	9,1	**0,002**
	14	9,7	10,2	10,4	**0,018**	9,0	9,7	9,6	**0,001**
	15	9,4	10,1	10,3	**0,000**	9,4	10,1	10,3	**0,000**
	16	8,4	9,7	10,0	**0,000**	9,2	10,2	10,7	**0,000**
	17	8,4	9,7	10,3	**0,000**	9,0	10,2	10,9	**0,000**
	18	8,9	10,1	10,5	**0,000**	9,1	10,5	11,1	**0,000**
	19	8,4	10,0	10,4	**0,000**	9,0	10,2	10,9	**0,000**
	20	8,0	9,2	9,9	**0,000**	8,5	9,6	10,3	**0,000**
	21	6,9	8,0	8,3	**0,000**	7,4	8,1	8,9	**0,000**
	22	5,8	6,6	7,0	**0,000**	6,5	7,4	7,5	**0,000**
	23	4,5	5,6	5,9	**0,000**	5,7	6,4	6,5	**0,000**
LPA minutes on weekdays (hourly average)	7	5,9	7,7	9,9	**0,355**	6,2	9,8	10,7	**0,006**
	8	12,8	14,3	13,4	**0,903**	14,1	13,6	14,5	**0,668**
	9	11,8	11,5	12,1	**0,581**	10,9	11,5	11,1	**0,072**
	10	9,9	9,6	9,5	**0,610**	8,0	8,9	8,8	**0,000**
	11	8,9	9,5	9,2	**0,147**	7,6	8,1	8,2	**0,002**
	12	9,3	9,3	9,6	**0,225**	7,7	8,5	8,7	**0,000**
	13	9,5	9,9	9,8	**0,154**	8,1	8,4	8,6	**0,091**
	14	10,1	10,2	10,3	**0,683**	8,8	9,5	9,2	**0,008**
	15	9,6	10,0	10,3	**0,013**	9,4	10,0	10,1	**0,000**
	16	8,5	9,4	9,8	**0,000**	9,2	10,1	10,7	**0,000**
	17	8,4	9,5	10,3	**0,000**	8,9	10,2	11,0	**0,000**
	18	8,9	10,1	10,7	**0,000**	9,2	10,7	11,3	**0,000**
	19	8,5	10,3	10,5	**0,000**	9,0	10,4	11,1	**0,000**
	20	8,1	9,4	10,0	**0,000**	8,7	9,8	10,6	**0,000**
	21	7,0	7,9	8,6	**0,000**	7,4	8,2	9,2	**0,000**
	22	5,8	6,7	7,0	**0,000**	6,3	7,4	7,7	**0,000**
	23	4,6	5,6	5,5	**0,042**	5,9	6,5	6,7	**0,000**
LPA minutes on weekends (hourly average)	7	2,0	2,7	2,1	**0,772**	2,0	3,7	2,9	**0,211**
	8	4,2	6,5	8,5	**0,007**	3,1	4,8	7,2	**0,002**
	9	6,8	7,2	10,5	**0,000**	6,7	7,9	9,2	**0,000**
	10	8,1	10,0	11,0	**0,000**	8,7	9,3	11,0	**0,000**
	11	9,5	9,6	11,1	**0,000**	10,0	10,2	12,0	**0,000**

12	9,0	10,4	11,2	**0,000**	9,9	10,9	11,4	**0,000**
13	8,7	10,0	10,1	**0,000**	9,7	11,0	11,0	**0,000**
14	8,6	10,0	10,6	**0,000**	9,9	10,2	11,1	**0,001**
15	8,9	10,4	10,6	**0,000**	9,7	10,6	10,9	**0,000**
16	8,3	10,7	10,6	**0,000**	9,5	10,5	10,8	**0,000**
17	8,7	10,5	10,5	**0,000**	9,6	10,4	10,6	**0,001**
18	8,6	10,0	10,0	**0,000**	8,8	9,9	10,4	**0,000**
19	8,1	9,0	9,8	**0,000**	8,7	9,5	10,1	**0,000**
20	7,7	8,5	9,2	**0,000**	7,7	8,8	9,4	**0,000**
21	6,8	8,1	7,5	**0,001**	7,3	7,7	8,2	**0,010**
22	5,9	6,6	6,7	**0,066**	6,5	7,6	7,2	**0,004**
23	4,4	6,0	5,7	**0,001**	5,2	7,1	6,5	**0,000**
