# Supplementary material for: Accelerometer-Measured Physical Activity Levels and Patterns Vary in an Age- and Sex-Dependent Fashion among Finnish Children and Adolescents
Source: Int J Environ Res Public Health. 2022 Jun 6;19(11):6950. doi: 10.3390/ijerph19116950 (PMC9180141; doi:10.3390/ijerph19116950)
Supplement: Supplementary file 1 [file ijerph-19-06950-s001.zip › TabS3. Participants hourly MET values by sex and tertiles and p-values for tertile differences.pdf]

Table S3. Participants' hourly MET values by sex and tertiles and p-values for tertile differences

		BOYS				GIRLS			
		step tertiles			**p-value (Kruskall-Wallis test)**	step tertiles			**p-value (Kruskall-Wallis test)**
	time of the day	least active	middle	most active		least active	middle	most active	
Maximum 1 minute MET (hourly average)	7	2,36	2,84	2,76	0,618	2,60	2,70	2,77	0,971
	8	4,19	4,31	4,76	0,006	4,10	4,32	4,44	0,020
	9	4,93	5,19	5,67	0,000	4,74	5,03	5,16	0,000
	10	5,18	5,64	6,12	0,000	4,68	5,02	5,26	0,000
	11	5,18	5,85	6,30	0,000	4,81	5,10	5,44	0,000
	12	5,44	6,18	6,74	0,000	4,94	5,32	5,73	0,000
	13	5,24	6,00	6,46	0,000	4,83	5,16	5,65	0,000
	14	5,00	5,67	6,18	0,000	4,60	5,11	5,52	0,000
	15	4,68	5,35	6,08	0,000	4,54	5,06	5,59	0,000
	16	4,18	5,13	5,79	0,000	4,29	4,79	5,42	0,000
	17	4,25	5,19	6,18	0,000	4,12	4,85	5,62	0,000
	18	4,46	5,60	6,65	0,000	4,30	5,07	6,02	0,000
	19	4,49	5,85	6,74	0,000	4,34	5,17	6,22	0,000
	20	4,35	5,61	6,51	0,000	4,35	5,18	5,94	0,000
	21	3,95	4,69	5,66	0,000	3,89	4,45	5,11	0,000
	22	3,45	3,98	4,62	0,000	3,51	3,97	4,42	0,000
	23	2,94	3,40	3,71	0,000	3,22	3,44	3,75	0,000
Maximum 1 minute MET on weekdays (hourly average)	7	2,49	3,04	2,83	0,977	2,69	2,92	3,02	0,593
	8	4,31	4,48	4,77	0,019	4,18	4,45	4,58	0,008
	9	5,04	5,39	5,70	0,000	4,82	5,10	5,26	0,000
	10	5,39	5,82	6,20	0,000	4,81	5,14	5,30	0,000
	11	5,43	6,12	6,37	0,000	4,95	5,22	5,46	0,000
	12	5,75	6,49	6,92	0,000	5,12	5,49	5,76	0,000
	13	5,55	6,25	6,59	0,000	5,02	5,33	5,64	0,000
	14	5,28	5,90	6,26	0,000	4,78	5,30	5,54	0,000
	15	4,87	5,43	6,03	0,000	4,66	5,17	5,62	0,000
	16	4,24	5,10	5,69	0,000	4,33	4,81	5,39	0,000
	17	4,29	5,25	6,20	0,000	4,14	4,91	5,67	0,000
	18	4,50	5,76	6,80	0,000	4,39	5,23	6,18	0,000
	19	4,55	6,03	6,94	0,000	4,45	5,30	6,43	0,000
	20	4,43	5,83	6,70	0,000	4,45	5,33	6,12	0,000
	21	4,01	4,81	5,83	0,000	3,89	4,53	5,27	0,000
	22	3,51	4,10	4,78	0,000	3,52	4,00	4,49	0,000
	23	3,01	3,47	3,72	0,000	3,31	3,57	3,94	0,000
Maximum 1 minute MET on weekends	7	1,93	2,13	2,20	0,542	1,79	1,92	2,18	0,766
	8	2,69	2,85	4,40	0,003	2,31	2,81	3,13	0,013
	9	3,47	3,69	5,06	0,000	3,27	3,84	4,12	0,000
	10	3,70	4,44	5,95	0,000	3,58	4,22	5,12	0,000

(hourly average)	11	4,00	4,66	6,25	0,000	4,03	4,57	5,39	0,000
	12	4,31	5,00	6,19	0,000	4,15	4,70	5,54	0,000
	13	4,16	5,15	6,01	0,000	4,07	4,57	5,64	0,000
	14	4,09	4,96	5,89	0,000	3,98	4,48	5,39	0,000
	15	4,12	5,14	6,19	0,000	4,13	4,70	5,45	0,000
	16	4,04	5,22	6,14	0,000	4,15	4,69	5,57	0,000
	17	4,16	5,06	6,18	0,000	4,13	4,66	5,48	0,000
	18	4,33	5,12	6,15	0,000	4,00	4,57	5,37	0,000
	19	4,27	5,27	6,07	0,000	4,04	4,66	5,52	0,000
	20	4,15	4,86	5,74	0,000	3,95	4,55	5,38	0,000
	21	3,78	4,36	5,22	0,000	3,90	4,17	4,55	0,001
	22	3,42	3,73	4,12	0,004	3,39	3,95	4,33	0,000
	23	2,85	3,43	3,54	0,000	3,04	3,39	3,54	0,000
